# The Evolving Landscape of COPD Typization

**DOI:** 10.3390/medicina62030564

**Published:** 2026-03-18

**Authors:** Alberto Fantin, Nadia Castaldo, Giulia Sartori, Claudia di Chiara, Filippo Patrucco, Giuseppe Morana, Vincenzo Patruno, Ernesto Crisafulli

**Affiliations:** 1 Department of Pulmonology, S. Maria della Misericordia University Hospital, 33100 Udine, Italy; 2 Department of Medicine, Respiratory Medicine Unit, University of Verona and Azienda Ospedaliera Universitaria Integrata of Verona, 37134 Verona, Italy; 3 Division of Respiratory Diseases, Department of Medicine, Maggiore della Carità University Hospital, 28100 Novara, Italy

**Keywords:** COPD, phenotype, endotype, eosinophil, omics

## Abstract

Chronic obstructive pulmonary disease (COPD) represents an escalating global health challenge characterized by profound clinical and biological heterogeneity. Conventional diagnostic paradigms, primarily reliant on spirometric criteria and broad phenotypic labels, often fail to capture the complex molecular mechanisms underlying effective precision medicine. This narrative review synthesizes the evolving landscape of COPD characterization, analyzing the integration of biomarkers, advanced quantitative imaging, and multi-omics technologies. Key developments highlighted include the clinical validation of biologics targeting type 2 inflammation, which reinforce the paradigm shift from generic symptomatic management toward the identification of specific treatable traits. We further explore the role of artificial intelligence and deep learning in enhancing radiological precision and body composition analysis. Ultimately, this work proposes a transition toward a GETomics (Genetics, Environment, and Time) framework as a fundamental prerequisite for transcending the limitations of traditional classification systems and delivering truly personalized care in the 21st century.

## 1. Introduction

Chronic obstructive pulmonary disease (COPD) remains a substantial and escalating global health challenge. Contemporary estimates indicate that the global prevalence of COPD among individuals aged 30–79 years is 10%, with 80% of cases residing in low and middle-income countries [1,2]. Projections suggest a further 23% increase in the number of cases by 2050, approaching 600 million worldwide, with the most marked growth anticipated among women and in lower-income settings [2]. Given the ageing population, persistent exposure to risk factors such as smoking, air pollution, and occupational hazards, COPD constitutes a major contributor to disability-adjusted life-years and mortality [3,4].

Despite its global importance, the current classification and diagnostic paradigm for COPD remain limited. Traditional frameworks, based primarily on spirometric criteria (such as post-bronchodilator FEV_1_/FVC < 0.70) and broad phenotypic labels, fail to capture the considerable heterogeneity in underlying molecular, structural, and functional mechanisms [5,6]. Moreover, these approaches provide limited prognostic discrimination and do not readily accommodate emerging technologies such as quantitative imaging, radiomics, multi-omics biomarkers, or artificial intelligence (AI)-driven phenotyping. Consequently, there is a pressing need to reconceptualize COPD classification and diagnostics to integrate these novel modalities, thereby enabling earlier detection, more accurate risk stratification, and personalized therapeutic strategies.

**Figure 1:**
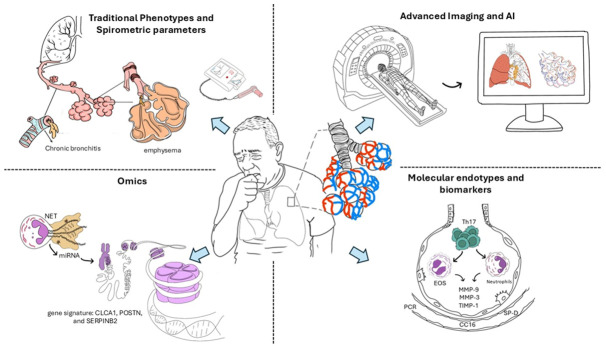
Integrative framework for the phenotypic and endotypic characterization of chronic obstructive pulmonary disease. AI: artificial intelligence.

In this narrative review, we aim to synthesize the evolving landscape of COPD management by elucidating how new technologies may be integrated into research and clinical practice (see Figure 1). We will explore the potential of these modalities to refine disease definitions, improve phenotyping, and advance precision medicine. Our scope encompasses the current evidence base, key technological innovations, and the translational challenges associated with bringing these developments into routine care. By highlighting the intersections between traditional clinical practice and emerging technology-enabled approaches, we aim to provide a forward-looking framework to support clinicians, researchers, and health system planners in addressing the complexities of COPD in the 21st century.

## 2. Methodology of the Review

Given the narrative nature of this review, the selection of references was primarily guided by the authors’ existing knowledge and familiarity with the literature. Relevant articles were identified based on their perceived significance, historical relevance, and practical impact on the topic under discussion, rather than through a systematic search strategy. Of note, the list of included references is not necessarily all-encompassing but reflects the body of evidence deemed appropriate to the purpose of this document: highlighting the integration of recent innovations in COPD research and clinical practice.

## 3. Heterogeneity of COPD: From Phenotype to Endotype

### 3.1. Classical Clinical Phenotypes

COPD has long been recognized as a clinically and biologically heterogeneous disorder. Traditional classification systems defined phenotypes according to dominant clinical and radiological manifestations, notably the *chronic* *bronchitic*, *emphysematous*, and *asthma–COPD overlap* (ACO) forms, each reflecting distinct symptom profiles, inflammatory patterns, and imaging correlates [7,8,9,10,11,12]. The chronic bronchitic phenotype, typified by cough and mucus hypersecretion, is associated with airway wall thickening, neutrophilic inflammation, and goblet cell hypertrophy. In contrast, the emphysematous phenotype is characterized by parenchymal destruction, loss of alveolar attachments, and hyperinflation, detectable by quantitative CT densitometry [13,14,15]. The ACO phenotype combines features of fixed functional obstruction and eosinophilic inflammation, frequently demonstrating greater corticosteroid responsiveness [16,17,18,19].

However, reliance on clinical and symptom-based distinctions provides only a partial representation of COPD’s biological diversity. Large imaging and transcriptomic consortia, such as COPDGene and SPIROMICS, have shown that radiological, inflammatory, and molecular features overlap extensively across classical clinical categories, challenging their specificity [20]. Furthermore, geographical and environmental variability, for instance, biomass exposure and post-tuberculosis airway injury in low- and middle-income countries, yield distinct phenotypic presentations characterized by less emphysema, greater air trapping, and heightened airway inflammation [21]. These findings underscore that the conventional phenotypes, though clinically pragmatic, do not sufficiently capture the mechanistic underpinnings of disease heterogeneity and its implications for precision medicine.

### 3.2. Emerging Molecular and Pathophysiological Endotypes

Recent advances have driven a fundamental reorientation from phenotype (see Table 1) to endotype, that is, to biologically defined disease subgroups sharing distinct molecular mechanisms (see Table 2). The integration of transcriptomic, proteomic, and metabolomic analyses with advanced radiomics and AI-based pattern recognition has revealed several reproducible endotypes. Collectively, these data highlight that COPD comprises a constellation of overlapping biological conditions rather than discrete entities.

**Figure 2:**
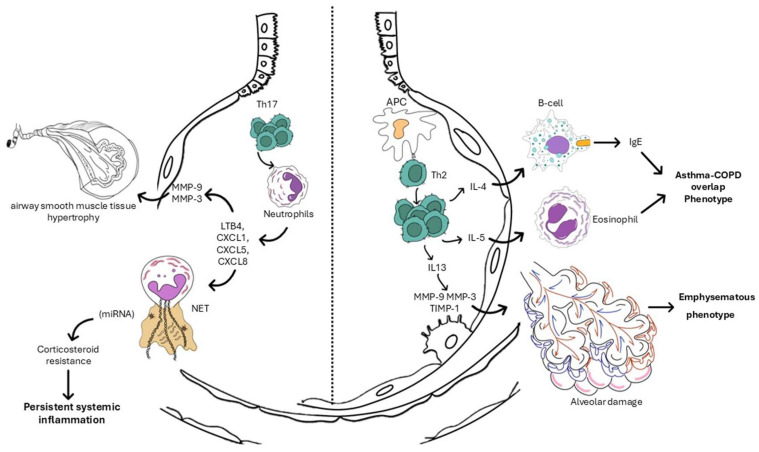
Pathophysiological mechanisms and inflammatory endotypes in chronic obstructive pulmonary disease.

The main COPD inflammatory endotypes are a type-2 eosinophilic phenotype, which remains corticosteroid-responsive despite frequent exacerbations [18,22], and a neutrophilic/Th17-dominant phenotype, characterized by refractory steroid resistance and chronic microbial colonization [23,24,25,26] (see Figure 2). Beyond inflammatory polarization, multi-omics profiling has identified distinct metabolic and transcriptomic signatures associated with tissue remodelling, mitochondrial dysfunction, and premature ageing, suggesting convergent pathways between COPD and other chronic systemic diseases such as heart failure (see the dedicated sections) [27,28,29,30,31,32].

Emerging evidence also supports exposure-related endotypes, including biomass-associated and post-tuberculosis COPD, which display unique airway remodelling patterns, elevated type-2 cytokines, and divergent imaging signatures compared with tobacco-related disease [21].

Furthermore, as delineated in Table 3, COPD exacerbations may be classified according to their biological etiology, primarily distinguishing between viral and bacterial phenotypes. This etiological stratification is pivotal for the implementation of precision therapeutic management, facilitating the adoption of antibiotic stewardship protocols and the targeted deployment of antivirals or immunomodulatory strategies [18,33,34].

## 4. Blood and Serum Biomarkers

The identification of blood-based biomarkers represents a cornerstone in the shift toward precision medicine for COPD. Historically viewed as a localized pulmonary condition, the current consensus, supported by extensive systemic analysis, recognizes COPD as a multicomponent disease associated with systemic inflammation [35,36,37,38].

### 4.1. Systemic Inflammatory Endotypes

C-reactive protein (CRP) and fibrinogen remain the most robustly validated systemic biomarkers for identifying the *persistent* *systemic inflammation* endotype. High baseline levels of these markers are associated with an increased risk of frequent exacerbations and all-cause mortality, even in patients with moderate airflow limitation [39,40,41]. Data from the SUMMIT trial demonstrated that, while these markers are predictive of clinical outcomes, they may not correlate with FEV1 decline rates in moderate stages, suggesting their primary utility lies in risk stratification rather than monitoring structural progression [41]. Furthermore, elevated fibrinogen levels have been identified as a distinguishing feature in specific overlap syndromes, such as the COPD-bronchiectasis overlap (BOS), where levels are significantly higher compared to pure COPD cohorts [42]. However, interpreting these biomarkers requires caution, as they can be influenced by comorbidities and other systemic factors [43].

### 4.2. Type 1 Inflammation and Neutrophilic Endotyping

The *neutrophilic endotype* (identified as a blood Neutrophil-to-Lymphocyte Ratio > 3.0) is the predominant inflammatory endotype in COPD, primarily driven by the activation of Type 1 (Th1) and Th17 immune pathways. This endotype is characteristically associated with corticosteroid resistance, chronic microbial colonization, and accelerated functional decline [44,45,46].

### 4.3. Type 2 Inflammation and Eosinophilic Endotyping

The typization of the *type 2 (T2)-high/eosinophilic* endotype (defined as ≥300 eosinophils/µL) using blood eosinophil counts represents the most tangible success of precision medicine in the COPD landscape. This endotype is no longer regarded as a mere variant of asthma but as a distinct pathophysiological driver associated with elevated Interleukin (IL)-4, IL-5, and IL-13 signaling pathways [18,19,20,47,48]. The blood eosinophil count serves as a surrogate for airway eosinophilia, identifying a subset of patients who exhibit a heightened response to inhaled corticosteroids (ICS) and targeted biological therapies [26,43]. This T2-high phenotype is increasingly viewed as a distinct clinical entity within the COPD landscape, necessitating regular eosinophil monitoring to optimize anti-inflammatory regimens [43].

### 4.4. Markers of Epithelial Injury and Lung Integrity

Recent advances have highlighted the prognostic value of serum proteins derived from the lung epithelium, such as Clara cell secretory protein (CC16) and Surfactant Protein D (SP-D). A deficiency in serum CC16 is a potent predictor of accelerated lung function decline and the development of early-onset COPD [49,50]. Conversely, elevated SP-D and surfactant protein A (SP-A) levels reflect increased alveolar-capillary permeability and are linked to disease severity and exacerbation frequency [51,52]. These markers provide a molecular window into the *fragile epithelium* endotype that precedes overt clinical deterioration.

### 4.5. Protease-Antiprotease Imbalance and Matrix Remodeling

The typization of COPD must account for the destructive processes of emphysema. Matrix metalloproteinases (notably MMP-9 and MMP-3) and their inhibitors (TIMP-1) are critical indicators of extracellular matrix remodeling. A high circulating MMP-9/TIMP-1 ratio and elevated serum MMP-3 have been identified as biomarkers for lung function decline and are particularly useful in differentiating ACO from pure asthma [35,53]. Additionally, novel peptides identified by mass spectrometry, such as histidine-rich glycoprotein (HRG) and alpha-1-acid glycoprotein (AGP1), offer high sensitivity for detecting chronic airflow limitation and outperform traditional single-marker assays [54].

### 4.6. Emerging Clustering Approaches

The future of COPD typization lies in clustering based on circulating biomarkers. Integrating inflammatory panels (IL-6, TNF-α, IL-18), injury markers (VEGF, ICAM-1), and serum metabolites (3-hydroxyisobutyrate, citrate) allows for the identification of distinct clusters, such as the *inflammatory-comorbid* cluster [55,56]. In fact, recent studies have also highlighted links between inflammatory biomarkers such as IL-18 and ICAM-1 and various comorbidities, suggesting a shared systemic inflammatory pathway [57]. These multi-marker signatures, visualized using methods such as the OSCAR plot, demonstrate that biomarker patterns better predict clinical outcomes than individual analytes [58].

## 5. Breathomics and Exhaled Breath Condensate

Breath analysis, encompassing both the fractional concentration of exhaled nitric oxide (FeNO) and the study of volatile organic compounds (VOCs), collectively termed breathomics, has emerged as a pivotal tool for identifying specific endotypes (e.g., T2-high) and predicting clinical trajectories [59,60].

### 5.1. FeNO and the T2-Inflammatory Endotype

While FeNO is traditionally associated with asthma, its role in COPD “FeNOtyping” is gaining traction as a biomarker for the T2-high treatable trait [61]. Elevated FeNO levels in COPD patients are indicative of eosinophilic airway inflammation and predict a favorable response to inhaled ICS [62]. However, the interpretation of FeNO in COPD is complex; cigarette smoking significantly downregulates NO synthase, often masking inflammatory signals in current smokers [63,64]. Recent efforts by the European Respiratory Society (ERS) and the Global Lung Function Initiative (GLI) have established new, robust reference values (2024) to standardize FeNO interpretation across different ages and demographics, aiming to reduce diagnostic ambiguity [65]. Furthermore, FeNO variability has been linked to exacerbation risk, suggesting its utility extends beyond static phenotyping to longitudinal monitoring [66].

### 5.2. Volatile Organic Compounds and Breathprints

Unlike FeNO, which measures a single molecule, breathomics analyzes the complex mixture of thousands of VOCs resulting from host metabolism and microbiome activity. This approach generates a breathprint that distinguishes COPD from healthy subjects with high accuracy [67,68]. Seminal work by Fens et al. demonstrated that electronic nose (eNose) technology could distinguish COPD from asthma with cross-validated accuracy exceeding 80%, independent of airway caliber [69]. This discriminative power has been further validated in recent studies using mass spectrometry (GC-MS) and sensor arrays to separate COPD from ACO, identifying specific metabolites such as fatty acids and valine as key discriminators [70].

Additionally, recent prospective studies have highlighted the eNose’s ability to stratify patients based on exacerbation phenotypes, distinguishing between bacterial and viral/non-infectious etiologies with high sensitivity [71,72]. Crucially, reproducibility studies in elderly COPD populations confirm that breath fingerprints remain stable over the short term in clinically stable patients, a prerequisite for longitudinal monitoring [73]. Moreover, the integration of Machine Learning algorithms, such as Adaptive Boosting and Support Vector Machines, has significantly enhanced the diagnostic accuracy of eNose systems, pushing diagnostic sensitivity toward 97–99% in controlled settings [74,75].

### 5.3. Emerging Frontiers

The landscape of breath analysis is evolving to address comorbidities and early disease states. The BreathCloud study provided compelling evidence that eNose technology can stratify lung cancer risk within the COPD population, offering a potential non-invasive screening tool for this high-risk group [76,77]. Additionally, 2025 data indicate that specific VOC patterns can identify the Preserved Ratio Impaired Spirometry (PRISm) pattern, suggesting that metabolic alterations may precede detectable spirometric decline [78].

### 5.4. Exhaled Breath Condensate

Complementing the gas phase, Exhaled Breath Condensate (EBC) offers a window into the liquid phase of the airway lining fluid. Markers of oxidative stress (e.g., 8-isoprostane) and pH levels in EBC have shown strong correlations with dyspnea scores and systemic inflammation, linking the biological endotype to the patient’s symptom burden [79].

## 6. Sputum Analysis

Notwithstanding the operational utility of systemic biomarkers, the analysis of induced or spontaneous sputum remains the reference standard for the direct evaluation of airway-specific inflammation, identifying biological endotypes that precipitate disease progression [80].

### 6.1. Granulocytic Typization

Cytometric analysis facilitates the stratification of COPD patients into four discrete inflammatory phenotypes: eosinophilic, neutrophilic, paucigranulocytic, and mixed granulocytic [81].

The *eosinophilic endotype* (conventionally defined by sputum eosinophilia ≥3%) epitomizes the T2-high endotype [82]. Crucially, recent data from the UCLA COPD Phenotyping Study have elucidated a significant discordance between the peripheral blood and sputum compartments [83]. LeMaster et al. demonstrated that exclusive reliance on blood eosinophil counts can lead to misclassification of airway inflammation in a substantial proportion of patients [84]. These findings reinforce sputum cytometry as the superior metric for confirming the T2-high trait, particularly when values are ambiguous or discordant with the clinical presentation.

The *neutrophilic endotype* (defined by sputum neutrophils >61%) shows, as well as the T2 one, a significant difference between circulating blood cell values and those measured in the airways [44,85,86]. This endotype is intrinsically associated with bacterial colonization and oxidative stress, characteristics that frequently correlate with the *frequent exacerbator* clinical phenotype [80,87]. Advancements in molecular diagnostics have revolutionized the management of this phenotype. Alotaibi et al. established that rapid polymerase chain reaction (PCR) assays on sputum samples can discriminate between bacterial and viral etiologies in hospitalized patients within hours, thereby facilitating precise antibiotic stewardship and mitigating inappropriate therapeutic interventions [34].

The *paucigranulocytic* endotype (conventionally defined by sputum neutrophils <61% and eosinophils <3%) represents a clinically stable yet biologically complex subset of the COPD population. In this endotype, airflow limitation is predominantly driven by structural alterations, such as small airway remodeling and loss of elastic recoil, rather than by active, protease-rich cellular recruitment [81]. While these patients often exhibit lower symptomatic burden and fewer exacerbations compared to their granulocytic counterparts, the paucigranulocytic state may also reflect a burnt-out inflammatory phase or the result of potent suppressive therapy [81]. Identifying this endotype is critical for therapeutic stewardship, as these patients typically derive minimal benefit from escalating anti-inflammatory regimens, including both corticosteroids and biologicals, suggesting that management should instead prioritize long-acting bronchodilators and non-pharmacological interventions like pulmonary rehabilitation [24,88].

The *mixed granulocytic* endotype (characterized by concurrent elevations of sputum neutrophils >61% and eosinophils ≥3%) represents the most severe and difficult-to-treat segment of the COPD spectrum [81]. This dual-pathway endotype signifies the simultaneous activation of Type 1/Th17 and T2 inflammatory cascades, resulting in an additive effect on tissue damage and symptom severity [18]. Clinical evidence indicates that patients with this endotype experience the highest frequency of exacerbations, the most rapid decline in lung function, and a significant degree of corticosteroid resistance despite the presence of eosinophilia [50,89]. The management of mixed granulocytic disease requires a sophisticated, multimodal approach, often combining targeted biological agents and macrolides to address the distinct yet overlapping drivers of airway injury [24,44,90,91,92].

### 6.2. The Microbiome-Proteome Interface

The integration of sputum microbiome interrogation has revealed that dysbiosis is a pivotal driver of the local inflammatory milieu. Multi-omic sputum analyses from the COPDMAP cohort have delineated distinct microbial clusters: a Haemophilus-predominant cluster, associated with neutrophilic inflammation and elevated exacerbation risk, and a Veillonella/Prevotella-predominant cluster, associated with a more stable clinical trajectory [93]. These microbial signatures are not merely epiphenomena but are actively associated with specific proteomic profiles implicated in host defense mechanisms and tissue destruction [93,94].

## 7. Bronchoscopic Retrieved Biomarkers

While sputum analysis characterizes the luminal inflammatory load, bronchoscopic sampling, encompassing bronchoalveolar lavage (BAL), endobronchial biopsies, and epithelial brushings, provides indispensable insight into the structural and distal airway compartments [95]. This invasive approach may be critical for resolving indeterminate typization attempts where non-invasive markers yield discordant or ambiguous results [50].

### 7.1. Tissue vs. Luminal Inflammation

Endobronchial biopsies allow for the quantification of tissue-resident inflammation and remodeling, which do not always mirror the luminal environment. Recent reviews highlight a frequent discordance between blood/sputum eosinophilia and tissue infiltration; Vanetti et al. recently emphasized that tissue-resident eosinophils are the primary drivers of airway remodeling via TGF-β release, a pathological feature often undetectable in sputum [96,97]. In difficult-to-treat COPD, histological assessment is uniquely capable of distinguishing active immune infiltration from fixed structural fibrosis, thereby guiding the appropriate de-escalation of steroid therapy in paucigranulocytic tissue endotypes [98].

### 7.2. Epithelial Transcriptomics

Beyond the inflammatory cell counts used in sputum analysis, bronchoscopic brushing interrogates the bronchial epithelium itself. Christenson et al. identified a robust Th2-high epithelial gene signature (including CLCA1, POSTN, and SERPINB2) in a subset of COPD patients [99]. Unlike the cellular signature derived from sputum granulocytes, this epithelial signature reflects the structural host response to inflammation and identifies patients with a T2-high epithelial endotype who benefit from targeted corticosteroid or biologic therapy, regardless of their clinical history [99,100].

### 7.3. The Lower Airway Microbiome

While sputum is prone to supraglottic contamination, bronchoscopic retrieval enables precise sampling of the distal lung microbiome. Segal and Huang have demonstrated that the specific enrichment of the lower airways with supraglottic bacteria (e.g., *Prevotella*, *Veillonella*) defines a *supraglottic-predominant* endotype linked to Th17 polarization [101]. This deep-lung sampling allows for the differentiation of pathogenic dysbiosis from mere oral contamination, offering a refined target for antimicrobial interventions in frequent exacerbators [101,102].

## 8. Advanced Imaging in COPD Characterization

The paradigm of radiological evaluation in COPD has undergone a fundamental transition from qualitative visual assessment to high-dimensional computational interrogation. Advanced imaging now serves as a non-invasive quantitative tool, enabling precise quantification of structural phenotypes, specifically distinguishing between airway-dominant and emphysema-dominant disease, and identifying early-stage precursors such as functional small airway disease (fSAD) [24,50,103].

### 8.1. Quantitative CT and the AI Revolution

The integration of AI and Deep Learning (DL) has revolutionized the precision of quantitative CT (QCT) and radiomics. Modern DL algorithms facilitate fully automated lung and lobe segmentation, providing objective staging and quantitation of emphysema and airway dynamics with a level of granularity previously unattainable [104,105,106]. Wu et al. (2024) highlighted that AI-powered insights into vascular structures and airway remodeling offer superior predictive value for clinical outcomes compared to traditional spirometric measures [105]. Furthermore, radiomic interrogation of bronchovascular bundle (BVB) textures has emerged as a radiological surrogate for systemic inflammation, bridging the gap between localized structural damage and systemic molecular activity [107,108].

### 8.2. Holistic Imaging Phenotyping: Body Composition and Comorbidities

Advanced imaging is increasingly utilized to characterize the multisystemic nature of COPD endotypes. Recent advancements include AI-based tools such as the Body and Organ Analysis (BOA) tool, which enables fully automated assessment of body composition from routine chest CT scans [109]. Budai et al. demonstrated that AI-derived metrics of sarcopenia and adipose tissue distribution correlate strongly with bioelectrical impedance analysis and serve as independent predictors of mortality and physical frailty in severe COPD, reinforcing the role of imaging in identifying the “cachectic” phenotype [109,110,111,112].

### 8.3. Parametric Response Mapping and the Small Airways

A significant challenge in COPD typization is the radiological identification of disease in the silent zone, the small airways (<2 mm). Parametric Response Mapping (PRM) co-registers inspiratory and expiratory scans to voxel-wise distinguish irreversible emphysema (PRMemph) from functional small airway disease (PRMfSAD) [113,114]. Recent longitudinal studies suggest that specific regional gas-trapping patterns are predictive of rapid FEV_1_ decline, identifying the *fast-progressor* phenotype before overt parenchymal destruction occurs [24,115].

### 8.4. Global Implementation and Early Screening

The clinical translation of AI-powered imaging is essential for addressing the global burden of COPD. Integrating AI into lung cancer screening protocols, particularly in primary healthcare settings, can enhance diagnostic accuracy and specificity for early-stage disease, including PRISm [116,117,118]. As emphasized by Robertson et al., the synergy between clinician expertise and AI analysis of large population-based cohorts is pivotal for moving beyond generic assessment tools toward a tailored medicine approach [117,119].

## 9. Omics

By analyzing genomic, transcriptomic, and metabolic layers, researchers have identified fundamental pathways that drive disease heterogeneity and treatment responsiveness [24,50].

### 9.1. Genomics and Known Genetic Variants

The genetic architecture of COPD extends far beyond the classical model of alpha-1-antitrypsin deficiency (AATD). Large-scale Genome-Wide Association Studies (GWAS) have identified over 80 loci associated with airflow limitation, with FAM13A and CHRNA3/5 being robustly linked to both emphysema and nicotine addiction [120,121].

The field is now advancing toward pharmacogenetics; Ntenti et al. have elucidated how genetic polymorphisms in metabolic enzymes and drug transporters (e.g., CYP2A6, ADRB2) shape individual responses to long-acting bronchodilators and corticosteroids, providing a blueprint for genetically tailored therapy [122]. Furthermore, Polygenic Risk Scores (PRS) are being refined to predict the development of a *fast-progressor* phenotype in asymptomatic smokers, identifying high-risk individuals before overt spirometric decline [28].

### 9.2. Transcriptomics and Gene Regulation

While genomics defines risk, transcriptomics captures the dynamic molecular state. Advanced RNA-sequencing has revealed increased transcript complexity in COPD, in which differential isoform usage, rather than total gene expression alone, drives disease pathology [123].

Beyond mRNA, microRNA(miRNA)-mRNA networks play a critical role in post-transcriptional regulation; Zhuang et al. identified specific miRNA clusters associated with systemic inflammation and structural remodeling in COPD [124]. At the single-cell level, transcriptomic profiling has identified a novel Neutrophil Extracellular Trap (NET) gene signature, which serves as a molecular surrogate for chronic neutrophilic inflammation and predicts exacerbation risk with higher capability than traditional cell counts and differential counts [125].

### 9.3. Proteomics and Metabolomics in Patient Characterization

High-throughput proteomics platforms (e.g., SOMAscan) have delineated distinct systemic clusters, some dominated by T2 markers (IL-5, CCL17) and others by neutrophilic mediators (MMP-9, IL-8), that correlate with mortality and structural damage [126,127].

Metabolomics further refines this typization by identifying sex-associated metabotypes. Naz et al. (2017) demonstrated that the autotaxin-lysoPA axis and oxidative stress pathways are differentially regulated in males and females, suggesting that precision medicine in COPD must account for sex-specific metabolic reprogramming [128]. Additionally, plasma metabolic signatures, characterized by dysregulated TCA cycle intermediates and branched-chain amino acids, have proven effective for identifying early-stage COPD in which routine blood tests remain unremarkable [129,130].

### 9.4. Epigenetics and Host-Microbe Interactions

Patients’ exposure exerts long-lasting effects through DNA methylation and histone modifications. Gao et al. highlighted that smoke-induced acetylation modifications are widely involved in airway remodelling and metabolic dysfunction [131].

Perhaps the most complex layer is the host-microbe interface. Yan et al. utilized sequential mediation analysis to demonstrate how microbial metabolites (e.g., short-chain fatty acids) interact with host gene expression to drive specific inflammatory endotypes, such as the *Th17-polarized* *neutrophilic* endotype [132]. This cross-kingdom endotyping reveals that the lung microbiome is not merely a bystander but an active driver of the host’s molecular trajectory [133].

## 10. Multimodal Integration: Towards Precision Medicine

This multimodal framework aims to identify treatable traits, clinical or biological attributes that can be specifically targeted to improve patient outcomes, reduce exacerbation frequency, and potentially modify the disease trajectory [24,89].

### 10.1. The Conceptual Reorientation

As emphasized by Christenson [120] and Corlateanu et al. [5], the precise characterization of underlying inflammatory pathways enables highly targeted interventions, ranging from the deployment of biological agents for the *T2-high* endotype to the strategic use of macrolides for neutrophilic or bacterial phenotypes [5,120]. Consequently, this taxonomic evolution necessitates moving beyond restrictive clinical assessment frameworks in favor of a biology-driven therapeutic strategy [5,120].

Agusti and Faner further expand this concept by introducing the GETomics model, a multi-layered approach that incorporates Genetics, Environment, and Time (age) [134]. This framework acknowledges that a patient’s phenotype is not a static state but a dynamic trajectome shaped by early-life factors and cumulative environmental exposures, collectively known as the exposome [134,135]. By integrating longitudinal data, clinicians can move toward preventive precision medicine, identifying individuals at high risk for rapid FEV_1_ decline before structural damage becomes irreversible and act earlier in the patient’s therapeutic pathway [135,136].

### 10.2. Technological Frontiers

Adding to the previous points, the ultimate goal of the Omics revolution is the synergistic integration of these layers. The development of Omic Risk Scores (ORS), which aggregate data from transcripts, proteins, and metabolites, is currently under investigation to evaluate their superiority over PRS alone for detecting the risk of emphysema progression and inducing rapid therapeutic and smoking cessation interventions [122,137].

Furthermore, AI and DL are now being used to discover hidden endotypes by linking molecular networks with clinical outcomes [138]. This integrated approach has already identified potential drug repurposing targets currently under evaluation, allowing researchers to match existing biologicals or small molecules to the specific molecular endotype of the patient, thereby progressively bridging the translational gap in COPD care [139,140].

The ability to integrate DL to acquire vast radiomic data from radiological images, as described by Wu and Segal, enables the identification of occult overlapping phenotypes and patterns, such as bronchiectasis and vascular pruning, which are critical traits for personalizing therapy in severe disease [141,142].

### 10.3. Clinical Validation and Implementation

Recent landmark trials have validated the clinical efficacy of this multimodal approach. The NOTUS and MATINEE studies provided definitive proof that targeting the *T2-high* endotype with biologics (Dupilumab and Mepolizumab, respectively) reduces exacerbations and improves quality of life in strictly defined high-eosinophilic populations [91,143].

Also, the *neutrophilic* endotype may be susceptible to targeted therapy. Emerging strategies now target this endotype through dipeptidyl peptidase-1 (DPP-1) inhibition (e.g., brensocatib), which prevents the activation of destructive proteases before they are released [44,144,145].

However, the challenge remains in integrating these high-dimensional datasets into routine clinical practice. van Zelst et al. highlighted that incorporating behavioral and psychosocial variables into cluster analyses creates more actionable real-world clinical profiles, ensuring that precision medicine addresses the patient as a whole rather than just a biological specimen [146]. As we move forward, the convergence of radiologic and molecular signatures associated with clinical treatable traits will define the standard of care for the evolving landscape of COPD [50,88].

## 11. Challenges and Future Perspectives

While the integration of multimodal data offers a transformative diagnostic framework, several translational barriers persist. There is a significant discrepancy between high-dimensional research findings and the resource-constrained environment of primary care (implementation gap). Future efforts must prioritize validating low-cost molecular and physiological surrogates that emulate the predictive power of expensive omics and quantitative CT [50].

The clinical utility of AI and digital typization depends on global standardization. Harmonizing protocols across diverse hardware platforms is essential to ensure that ORS and topological imaging metrics are reproducible and reliable [147,148].

Identifying PRISm remains an unmet priority. Longitudinal GETomics studies are needed to pinpoint the biological triggers that convert these early trajectories into irreversible airflow limitation [134].

Current Phase 3 evidence is heavily skewed toward tobacco-related COPD. Future typization must incorporate non-tobacco phenotypes, driven by biomass exposure and air pollution, to address the escalating global burden of the disease [149].

## 12. Conclusions

The landscape of COPD characterization has reached a pivotal juncture. Clinical labels such as chronic bronchitis and emphysema are no longer sufficient; they are now recognized as the macroscopic consequences of diverse molecular endotypes. The successful clinical validation of biologics targeting the T2-high endotype, demonstrated by the NOTUS and MATINEE trials, provides definitive proof that precisely identifying a treatable trait via multimodal integration yields superior outcomes.

Ultimately, the transition toward a GETomics framework, incorporating genomic risk, environment, and time, enables a move beyond generic spirometric classification. This biology-driven conceptual evolution is the fundamental prerequisite for reducing global COPD morbidity and delivering truly personalized care in the 21st century.

## Figures and Tables

**Table 1:** COPD phenotypes.

Phenotype	Characteristics
Chronic Bronchitic	The presence of a productive cough for more than 3 months per year in two or more consecutive years
Emphysematous	Prevalent emphysema pattern on chest CT
Frequent exacerbator	Two or more exacerbations per year
Rare exacerbator	None or rare (1 per year) exacerbations
Asthma-COPD-Overlap	Persistent airflow limitation. Clinical and functional features of both asthma and COPD
Bronchiectasis-COPD-Overlap	Prevalent bronchiectasis pattern on chest CT
Pulmonary cachexia phenotype	Low Body Mass Index
Upper lobe-predominant Emphysema	Upper lobes emphysema pattern on chest CT
Fast decliner phenotype	Decline of lung function above 60 mL of FEV_1_ per year
The comorbid or systemic phenotype	High number of comorbidities
Non-smoker COPD	Younger age, higher BMI, female predominance, less emphysema, greater small airway disease, frequent biomass/pollution exposure, and potentially more comorbidities (asthma, TB, bronchiectasis).

**Table 2:** COPD endotypes.

Endotype	Characteristics
α1-Antitrypsin Deficiency	Precocious emphysema. Reduced plasma alpha-1-antitrypsin
Telomerase polymorphisms	TERT polymorphism. Reduced telomere length.
Persistent systemic inflammation	Consistently high inflammation markers
Eosinophilic	High blood or sputum eosinophils
Neutrophilic	High blood or sputum neutrophils
Paucigranulocytic	Low levels of both neutrophils and eosinophils in the sputum
Mixed granulocytic	Simultaneous elevation of both neutrophils and eosinophils in sputum or blood

**Table 3:** Exacerbation phenotypes.

Type	Characteristics
Viral	Increased sputum CXCL10, identified viral etiology (e.g., nasal swab, alveolar lavage)
Bacterial	Sputum purulence, identified bacterial etiology (e.g., nasal swab, bronchoalveolar lavage, urinary antigen)

## Data Availability

No new data were created or analyzed in this study.

## Abbreviations

The following abbreviations are used in this manuscript:

AATD	Alpha-1-Antitrypsin Deficiency
ACO	Asthma–COPD Overlap
ADRB2	Adrenoceptor Beta 2
AGP1	Alpha-1-acid glycoprotein
AI	Artificial Intelligence
BAL	Bronchoalveolar Lavage
BOA	Body and Organ Analysis
BOS	COPD-bronchiectasis overlap syndrome
COPD	Chronic Obstructive Pulmonary Disease
CRP	C-reactive protein
CT	Computed Tomography
CYP2A6	Cytochrome P450 2A6
DL	Deep Learning
DPP-1	Dipeptidyl peptidase-1
EBC	Exhaled Breath Condensate
eNose	Electronic Nose
ERS	European Respiratory Society
FeNO	Fractional concentration of exhaled nitric oxide
FEV_1_	Forced Expiratory Volume in 1 s
fSAD	functional Small Airway Disease
FVC	Forced Vital Capacity
GC-MS	Gas Chromatography-Mass Spectrometry
GETomics	Genetics, Environment, and Time
GLI	Global Lung Function Initiative
GWAS	Genome-Wide Association Studies
HRG	Histidine-rich glycoprotein
ICAM-1	Intercellular Adhesion Molecule 1
ICS	Inhaled Corticosteroids
IL	Interleukin
miRNA	Micro RNA
MMP	Matrix Metalloproteinase
mRNA	Messenger RNA
NET	Neutrophil Extracellular Trap
ORS	Omic Risk Score
PCR	Polymerase Chain Reaction
PRISm	Preserved Ratio Impaired Spirometry
PRM	Parametric Response Mapping
PRS	Polygenic Risk Score
QCT	Quantitative Computed Tomography
SP-A/SP-D	Surfactant Protein A/D
T2	Type 2 inflammation
TB	Tuberculosis
TGF-β	Transforming Growth Factor beta
TIMP-1	Tissue Inhibitor of Metalloproteinases 1
TNF-α	Tumor Necrosis Factor alpha
VEGF	Vascular Endothelial Growth Factor
VOCs	Volatile Organic Compounds

## References

[1] Adeloye D., Song P., Zhu Y., Campbell H., Sheikh A., Rudan I. (2022). NIHR RESPIRE Global Respiratory Health Unit Global, Regional, and National Prevalence of, and Risk Factors for, Chronic Obstructive Pulmonary Disease (COPD) in 2019: A Systematic Review and Modelling Analysis. Lancet Respir. Med..

[2] Boers E., Barrett M., Su J.G., Benjafield A.V., Sinha S., Kaye L., Zar H.J., Vuong V., Tellez D., Gondalia R. (2023). Global Burden of Chronic Obstructive Pulmonary Disease Through 2050. JAMA Netw. Open.

[3] Zou J., Sun T., Song X., Liu Y.-M., Lei F., Chen M.-M., Chen Z., Zhang P., Ji Y.-X., Zhang X.-J. (2022). Distributions and Trends of the Global Burden of COPD Attributable to Risk Factors by SDI, Age, and Sex from 1990 to 2019: A Systematic Analysis of GBD 2019 Data. Respir. Res..

[4] Eisner M.D., Iribarren C., Blanc P.D., Yelin E.H., Ackerson L., Byl N., Omachi T.A., Sidney S., Katz P.P. (2011). Development of Disability in Chronic Obstructive Pulmonary Disease: Beyond Lung Function. Thorax.

[5] Corlateanu A., Mendez Y., Wang Y., de Jesus Avendaño Garnicad R., Botnaru V., Siafakas N. (2020). Chronic Obstructive Pulmonary Disease and Phenotypes: A State-of-the-Art. Pulmonology.

[6] Agustí A., Celli B.R., Criner G.J., Halpin D., Anzueto A., Barnes P., Bourbeau J., Han M.K., Martinez F.J., Montes de Oca M. (2023). Global Initiative for Chronic Obstructive Lung Disease 2023 Report: GOLD Executive Summary. Eur. Respir. J..

[7] Miravitlles M., Soler-Cataluña J.J., Calle M., Soriano J.B. (2013). Treatment of COPD by Clinical Phenotypes: Putting Old Evidence into Clinical Practice. Eur. Respir. J..

[8] Fragoso E., André S., Boleo-Tomé J.P., Areias V., Munhá J., Cardoso J. (2016). Understanding COPD: A Vision on Phenotypes, Comorbidities and Treatment Approach. Rev. Port. Pneumol..

[9] Crisafulli E., Guerrero M., Ielpo A., Ceccato A., Huerta A., Gabarrús A., Soler N., Chetta A., Torres A. (2018). Impact of Bronchiectasis on Outcomes of Hospitalized Patients with Acute Exacerbation of Chronic Obstructive Pulmonary Disease: A Propensity Matched Analysis. Sci. Rep..

[10] Bakeer M., Funk G.-C., Valipour A. (2020). Chronic Obstructive Pulmonary Disease Phenotypes: Imprint on Pharmacological and Non-Pharmacological Therapy. Ann. Transl. Med..

[11] Rinaldo R.F., Mondoni M., Comandini S., Lombardo P., Vigo B., Terraneo S., Santus P., Carugo S., Centanni S., Marco F.D. (2020). The Role of Phenotype on Ventilation and Exercise Capacity in Patients Affected by COPD: A Retrospective Study. Multidiscip. Respir. Med..

[12] Fortis S., Georgopoulos D., Tzanakis N., Sciurba F., Zabner J., Comellas A.P. (2024). Chronic Obstructive Pulmonary Disease (COPD) and COPD-like Phenotypes. Front. Med..

[13] Kim V., Criner G.J. (2013). Chronic Bronchitis and Chronic Obstructive Pulmonary Disease. Am. J. Respir. Crit. Care Med..

[14] Washko G.R. (2010). Diagnostic Imaging in COPD. Semin. Respir. Crit. Care Med..

[15] Tanabe N., Nakagawa H., Sakao S., Ohno Y., Shimizu K., Nakamura H., Hanaoka M., Nakano Y., Hirai T. (2024). Lung Imaging in COPD and Asthma. Respir. Investig..

[16] Calverley P.M.A., Walker P.P. (2021). ACO (Asthma–COPD Overlap) Is Independent from COPD: The Case Against. Diagnostics.

[17] Fouka E., Papaioannou A.I., Hillas G., Steiropoulos P. (2022). Asthma-COPD Overlap Syndrome: Recent Insights and Unanswered Questions. J. Pers. Med..

[18] Bhatt S.P., Agusti A., Bafadhel M., Christenson S.A., Bon J., Donaldson G.C., Sin D.D., Wedzicha J.A., Martinez F.J. (2023). Phenotypes, Etiotypes, and Endotypes of Exacerbations of Chronic Obstructive Pulmonary Disease. Am. J. Respir. Crit. Care Med..

[19] Alsayed A.R., Abu-Samak M.S., Alkhatib M. (2023). Asthma-COPD Overlap in Clinical Practice (ACO_CP 2023): Toward Precision Medicine. J. Pers. Med..

[20] Bell A.J., Ram S., Labaki W.W., Murray S., Kazerooni E.A., Galban S., Martinez F.J., Hatt C.R., Wang J.M., Ivanov V. (2025). Temporal Exploration of Chronic Obstructive Pulmonary Disease Phenotypes: Insights from the COPDGene and SPIROMICS Cohorts. Am. J. Respir. Crit. Care Med..

[21] Siddharthan T., Gupte A., Barnes P.J. (2020). Chronic Obstructive Pulmonary Disease Endotypes in Low- and Middle-Income Country Settings: Precision Medicine for All. Am. J. Respir. Crit. Care Med..

[22] Barnes P.J. (2019). Inflammatory Endotypes in COPD. Allergy.

[23] Rouzic O.L., Pichavant M., Frealle E., Guillon A., Si-Tahar M., Gosset P. (2017). Th17 Cytokines: Novel Potential Therapeutic Targets for COPD Pathogenesis and Exacerbations. Eur. Respir. J..

[24] Xie C., Wang K., Yang K., Zhong Y., Gul A., Luo W., Yalikun M., He J., Chen W., Xu W. (2025). Toward Precision Medicine in COPD: Phenotypes, Endotypes, Biomarkers, and Treatable Traits. Respir. Res..

[25] Lourenço J.D., Ito J.T., Martins M.d.A., Tibério I.d.F.L.C., Lopes F.D.T.Q.d.S. (2021). Th17/Treg Imbalance in Chronic Obstructive Pulmonary Disease: Clinical and Experimental Evidence. Front. Immunol..

[26] Brightling C., Greening N. (2019). Airway Inflammation in COPD: Progress to Precision Medicine. Eur. Respir. J..

[27] Li H., Huang C., Su R., Wang M., Ma Y., Wang Y., Xu B., Liu K. (2025). Developing a Panel of Shared Susceptibility Genes as Diagnostic Biomarkers for Chronic Obstructive Pulmonary Disease and Heart Failure. Comput. Biol. Med..

[28] Regan E.A., Hersh C.P., Castaldi P.J., DeMeo D.L., Silverman E.K., Crapo J.D., Bowler R.P. (2019). Omics and the Search for Blood Biomarkers in Chronic Obstructive Pulmonary Disease. Insights from COPDGene. Am. J. Respir. Cell Mol. Biol..

[29] Crisafulli E., Sartori G., Vianello A., Busti F., Nobili A., Mannucci P.M., Girelli D. (2023). REPOSI Investigators Clinical Features and Outcomes of Elderly Hospitalised Patients with Chronic Obstructive Pulmonary Disease, Heart Failure or Both. Intern. Emerg. Med..

[30] Li C.-L., Liu J.-F., Liu S.-F. (2024). Mitochondrial Dysfunction in Chronic Obstructive Pulmonary Disease: Unraveling the Molecular Nexus. Biomedicines.

[31] Abdellaoui A., Gouzi F., Notarnicola C., Bourret A., Suc A., Laoudj-Chenivesse D., Héraud N., Mercier J., Préfaut C., Hayot M. (2025). Mitochondrial Dysfunction and Defects in Mitochondrial Adaptation to Exercise Training in the Muscle of Patients With COPD: Disease Versus Disuse. Acta Physiol..

[32] Rabinovich R.A., Bastos R., Ardite E., Llinàs L., Orozco-Levi M., Gea J., Vilaró J., Barberà J.A., Rodríguez-Roisin R., Fernández-Checa J.C. (2007). Mitochondrial Dysfunction in COPD Patients with Low Body Mass Index. Eur. Respir. J..

[33] Bafadhel M., McKenna S., Terry S., Mistry V., Reid C., Haldar P., McCormick M., Haldar K., Kebadze T., Duvoix A. (2011). Acute Exacerbations of Chronic Obstructive Pulmonary Disease: Identification of Biologic Clusters and Their Biomarkers. Am. J. Respir. Crit. Care Med..

[34] Alotaibi N.M., Chen V., Hollander Z., Leipsic J.A., Hague C.J., Murphy D.T., DeMarco M.L., FitzGerald J.M., McManus B.M., Ng R.T. (2019). Phenotyping and Outcomes of Hospitalized COPD Patients Using Rapid Molecular Diagnostics on Sputum Samples. Int. J. Chronic Obs. Pulm. Dis..

[35] Higashimoto Y., Iwata T., Okada M., Satoh H., Fukuda K., Tohda Y. (2009). Serum Biomarkers as Predictors of Lung Function Decline in Chronic Obstructive Pulmonary Disease. Respir. Med..

[36] Hizawa N. (2012). Associating Serum Biomarkers with Genetic Susceptibility to Chronic Obstructive Pulmonary Disease: A Step towards Improved Diagnosis and Therapy?. Am. J. Respir. Crit. Care Med..

[37] Fattouh M., Alkady O. (2014). Inflammatory Biomarkers in Chronic Obstructive Pulmonary Disease. Egypt. J. Chest Dis. Tuberc..

[38] Crisafulli E., Manco A., Ferrer M., Huerta A., Micheletto C., Girelli D., Clini E., Torres A. (2020). Pneumonic versus Nonpneumonic Exacerbations of Chronic Obstructive Pulmonary Disease. Semin. Respir. Crit. Care Med..

[39] Duvoix A., Dickens J., Haq I., Mannino D., Miller B., Tal-Singer R., Lomas D.A. (2013). Blood Fibrinogen as a Biomarker of Chronic Obstructive Pulmonary Disease. Thorax.

[40] Crisafulli E., Torres A., Huerta A., Méndez R., Guerrero M., Martinez R., Liapikou A., Soler N., Sethi S., Menéndez R. (2015). C-Reactive Protein at Discharge, Diabetes Mellitus and ≥1 Hospitalization During Previous Year Predict Early Readmission in Patients with Acute Exacerbation of Chronic Obstructive Pulmonary Disease. COPD J. Chronic Obstr. Pulm. Dis..

[41] Celli B.R., Anderson J.A., Brook R., Calverley P., Cowans N.J., Crim C., Dixon I., Kim V., Martinez F.J., Morris A. (2019). Serum Biomarkers and Outcomes in Patients with Moderate COPD: A Substudy of the Randomised SUMMIT Trial. BMJ Open Respir. Res..

[42] Sever Z.K., Bircan H.A., Sirin F.B., Evrimler S., Celik S., Merd N. (2020). Serum Biomarkers in Patients with Stable and Exacerbated COPD-Bronchiectasis Overlap Syndrome. Clin. Respir. J..

[43] Stockley R.A., Halpin D.M.G., Celli B.R., Singh D. (2019). Chronic Obstructive Pulmonary Disease Biomarkers and Their Interpretation. Am. J. Respir. Crit. Care Med..

[44] Fricker M., Lokwani R. (2025). COPD: The Role of Neutrophils in Inflammation, Pathophysiology, and as Drug Targets. Clin. Sci..

[45] Hoenderdos K., Condliffe A. (2013). The Neutrophil in Chronic Obstructive Pulmonary Disease. Am. J. Respir. Cell Mol. Biol..

[46] Mariotti B., Bracaglia C., Gasperini S., Sartori G., Crisafulli E., Bazzoni F. (2025). Innate Immune Reprogramming in Circulating Neutrophils of COPD Patients. J. Allergy Clin. Immunol..

[47] Ferrari M., Pizzini M., Cazzoletti L., Ermon V., Spelta F., De Marchi S., Carbonare L.G.D., Crisafulli E. (2022). Circulating Eosinophil Levels and Lung Function Decline in Stable Chronic Obstructive Pulmonary Disease: A Retrospective Longitudinal Study. J. Bras. Pneumol..

[48] Polverino F., Sin D.D. (2024). Type 2 Airway Inflammation in COPD. Eur. Respir. J..

[49] Shaw J.G., Vaughan A., Dent A.G., O’Hare P.E., Goh F., Bowman R.V., Fong K.M., Yang I.A. (2014). Biomarkers of Progression of Chronic Obstructive Pulmonary Disease (COPD). J. Thorac. Dis..

[50] Phillips K.M., Lavere P.F., Hanania N.A., Adrish M. (2025). The Emerging Biomarkers in Chronic Obstructive Pulmonary Disease: A Narrative Review. Diagnostics.

[51] Dahl M. (2008). Biomarkers for Chronic Obstructive Pulmonary Disease: Surfactant Protein D and C-Reactive Protein. Am. J. Respir. Crit. Care Med..

[52] Ambade V.N. (2016). Serum Biomarkers of COPD—Reply. Respiratory Care.

[53] Takada K., Suzukawa M., Tashimo H., Ohshima N., Fukutomi Y., Kobayashi N., Taniguchi M., Ishii M., Akishita M., Ohta K. (2023). Serum MMP3 and IL1-RA Levels May Be Useful Biomarkers for Detecting Asthma and Chronic Obstructive Pulmonary Disease Overlap in Patients with Asthma. World Allergy Organ. J..

[54] Molin M., Incamps A., Lemasson M., Andersson M., Pertsinidou E., Högman M., Lisspers K., Ställberg B., Sjölander A., Malinovschi A. (2024). Biomarkers of Chronic Airflow Limitation and COPD Identified by Mass Spectrometry. ERJ Open Res..

[55] Vanfleteren L.E.G.W., Weidner J., Franssen F.M.E., Gaffron S., Reynaert N.L., Wouters E.F.M., Spruit M.A. (2023). Biomarker-Based Clustering of Patients with Chronic Obstructive Pulmonary Disease. ERJ Open Res..

[56] Chen Q., Deeb R.S., Ma Y., Staudt M.R., Crystal R.G., Gross S.S. (2015). Serum Metabolite Biomarkers Discriminate Healthy Smokers from COPD Smokers. PLoS ONE.

[57] Małujło-Balcerska E., Pietras T., Śmigielski W. (2023). Serum Levels of Biomarkers That May Link Chronic Obstructive Pulmonary Disease and Depressive Disorder. Pharmacol. Rep..

[58] Pinto-Plata V., Casanova C., Müllerova H., de Torres J.P., Corado H., Varo N., Cordoba E., Zeineldine S., Paz H., Baz R. (2012). Inflammatory and Repair Serum Biomarker Pattern. Association to Clinical Outcomes in COPD. Respir. Res..

[59] Santini G., Mores N., Penas A., Capuano R., Mondino C., Trové A., Macagno F., Zini G., Cattani P., Martinelli E. (2016). Electronic Nose and Exhaled Breath NMR-Based Metabolomics Applications in Airways Disease. Curr. Top. Med. Chem..

[60] Bos L.D., Sterk P.J., Fowler S.J. (2016). Breathomics in the Setting of Asthma and Chronic Obstructive Pulmonary Disease. J. Allergy Clin. Immunol..

[61] Flynn C., Brightling C. (2023). Is FeNOtyping in COPD the Path to Precision Medicine?. Respirology.

[62] Ragnoli B., Radaeli A., Pochetti P., Kette S., Morjaria J., Malerba M. (2023). Fractional Nitric Oxide Measurement in Exhaled Air (FeNO): Perspectives in the Management of Respiratory Diseases. Ther. Adv. Chronic Dis..

[63] Dweik R.A., Boggs P.B., Erzurum S.C., Irvin C.G., Leigh M.W., Lundberg J.O., Olin A.-C., Plummer A.L., Taylor D.R. (2011). American Thoracic Society Committee on Interpretation of Exhaled Nitric Oxide Levels (FENO) for Clinical Applications An Official ATS Clinical Practice Guideline: Interpretation of Exhaled Nitric Oxide Levels (FENO) for Clinical Applications. Am. J. Respir. Crit. Care Med..

[64] Högman M., Thornadtsson A., Bröms K., Janson C., Lisspers K., Ställberg B., Hedenström H., Malinovschi A. (2019). Different Relationships between FENO and COPD Characteristics in Smokers and Ex-Smokers. COPD.

[65] Högman M., Bowerman C., Chavez L., Dressel H., Malinovschi A., Radtke T., Stanojevic S., Steenbruggen I., Turner S., Dinh-Xuan A.T. (2024). ERS Technical Standard: Global Lung Function Initiative Reference Values for Exhaled Nitric Oxide Fraction (F ENO50). Eur. Respir. J..

[66] Ambrosino P., Fuschillo S., Accardo M., Mosella M., Molino A., Spedicato G.A., Motta A., Maniscalco M. (2022). Fractional Exhaled Nitric Oxide (FeNO) in Patients with Stable Chronic Obstructive Pulmonary Disease: Short-Term Variability and Potential Clinical Implications. J. Pers. Med..

[67] Besa V., Teschler H., Kurth I., Khan A.M., Zarogoulidis P., Baumbach J.I., Sommerwerck U., Freitag L., Darwiche K. (2015). Exhaled Volatile Organic Compounds Discriminate Patients with Chronic Obstructive Pulmonary Disease from Healthy Subjects. Int. J. Chronic Obs. Pulm. Dis..

[68] Jareño-Esteban J.J., Muñoz-Lucas M.Á., Gómez-Martín Ó., Utrilla-Trigo S., Gutiérrez-Ortega C., Aguilar-Ros A., Collado-Yurrita L., Callol-Sánchez L.M. (2017). Study of 5 Volatile Organic Compounds in Exhaled Breath in Chronic Obstructive Pulmonary Disease. Arch. Bronconeumol..

[69] Fens N., Zwinderman A.H., van der Schee M.P., de Nijs S.B., Dijkers E., Roldaan A.C., Cheung D., Bel E.H., Sterk P.J. (2009). Exhaled Breath Profiling Enables Discrimination of Chronic Obstructive Pulmonary Disease and Asthma. Am. J. Respir. Crit. Care Med..

[70] Ghosh N., Choudhury P., Joshi M., Bhattacharyya P., Roychowdhury S., Banerjee R., Chaudhury K. (2021). Global Metabolome Profiling of Exhaled Breath Condensates in Male Smokers with Asthma COPD Overlap and Prediction of the Disease. Sci. Rep..

[71] Shafiek H., Fiorentino F., Merino J.L., López C., Oliver A., Segura J., de Paul I., Sibila O., Agustí A., Cosío B.G. (2015). Using the Electronic Nose to Identify Airway Infection during COPD Exacerbations. PLoS ONE.

[72] van Bragt J.J.M.H., Brinkman P., de Vries R., Vijverberg S.J.H., Weersink E.J.M., Haarman E.G., de Jongh F.H.C., Kester S., Lucas A., In ’t Veen J.C.C.M. (2020). Identification of Recent Exacerbations in COPD Patients by Electronic Nose. ERJ Open Res..

[73] Incalzi R.A., Pennazza G., Scarlata S., Santonico M., Petriaggi M., Chiurco D., Pedone C., Arnaldo, D’Amico (2012). Reproducibility and Respiratory Function Correlates of Exhaled Breath Fingerprint in Chronic Obstructive Pulmonary Disease. PLoS ONE.

[74] Binson V.A., Subramoniam M., Mathew L. (2021). Detection of COPD and Lung Cancer with Electronic Nose Using Ensemble Learning Methods. Clin. Chim. Acta.

[75] Aulia D., Rivai M., Aulia S., Nur A.F. (2025). Non-Invasive Exhaled Breath Analysis for Chronic Obstructive Pulmonary Disease Classification Using an Electronic Nose System. Procedia Comput. Sci..

[76] de Vries R., Farzan N., Fabius T., De Jongh F.H.C., Jak P.M.C., Haarman E.G., Snoey E., In ’t Veen J.C.C.M., Dagelet Y.W.F., Maitland-Van Der Zee A.-H. (2023). Prospective Detection of Early Lung Cancer in Patients With COPD in Regular Care by Electronic Nose Analysis of Exhaled Breath. Chest.

[77] Satici C., Bek E.O. (2024). The Role of Electronic Nose Analysis of Exhaled Air in Detection of Lung Cancer Among Patients with COPD. Chest.

[78] Tian J., Zhang Q., Peng M., Guo L., Zhao Q., Lin W., Chen S., Liu X., Xie S., Wu W. (2025). Exhaled Volatile Organic Compounds as Novel Biomarkers for Early Detection of COPD, Asthma, and PRISm: A Cross-Sectional Study. Respir. Res..

[79] Patsiris S., Stelios G., Papanikolaou I., Exarchos T., Vlamos P. (2021). Exhaled Breath Condensate: A Potential Tool to Evaluate the Relationship between Inflammation and Dyspnea in Patients with Chronic Obstructive Pulmonary Disease. J. Lung Health Dis..

[80] Miravitlles M., Calle M., Soler-Cataluña J.J. (2012). Clinical Phenotypes of COPD: Identification, Definition and Implications for Guidelines. Arch. Bronconeumol..

[81] Wen X., Deng Z., Peng J., Yang H., Wu F., Dai C., Zheng Y., Zhao N., Wang Z., Xiao S. (2023). Characteristics of Inflammatory Phenotypes in Patients with Chronic Obstructive Pulmonary Disease: A Cross-Sectional Study. BMJ Open Respir. Res..

[82] Bartoli M.L., Latorre M., Vagaggini B., Nieri D., Cianchetti S., Di Franco A., Celi A., Paggiaro P. (2021). Are Sputum Eosinophils Associated with a Different Phenotype in COPD Patients? A Retrospective Study. Respir. Med..

[83] Fat M., Andersen T., Fazio J.C., Park S.C., Abtin F., Buhr R.G., Phillips J.E., Belperio J., Tashkin D.P., Cooper C.B. (2024). Association of Bronchial Disease on CT Imaging and Clinical Definitions of Chronic Bronchitis in a Single-Center COPD Phenotyping Study. Respir. Med..

[84] LeMaster W.B., Ingersoll S.A., Phee H., Wen R., Bai J., Belperio J.A., Buhr R.G., Phillips J.E., Palchevskiy V., Bina T. (2025). Diagnosing Type 2 Inflammation in COPD: Comparison of Blood and Sputum Eosinophil Assessment in the University of California Los Angeles COPD Phenotyping Study. Chronic Obstr. Pulm. Dis..

[85] Singh D., Edwards L., Tal-Singer R., Rennard S. (2010). Sputum Neutrophils as a Biomarker in COPD: Findings from the ECLIPSE Study. Respir. Res..

[86] Gupta V., Singh D. (2013). Critical Assessment of the Value of Sputum Neutrophils. COPD: J. Chronic Obstr. Pulm. Dis..

[87] Yang C.-Y., Li S.-W., Chin C.-Y., Hsu C.-W., Lee C.-C., Yeh Y.-M., Wu K.-A. (2021). Association of Exacerbation Phenotype with the Sputum Microbiome in Chronic Obstructive Pulmonary Disease Patients during the Clinically Stable State. J. Transl. Med..

[88] Wouters E.F.M., Wouters B.B.R.E.F., Augustin I.M.L., Houben-Wilke S., Vanfleteren L.E.G.W., Franssen F.M.E. (2018). Personalised Pulmonary Rehabilitation in COPD. Eur. Respir. Rev..

[89] Agusti A., Bel E., Thomas M., Vogelmeier C., Brusselle G., Holgate S., Humbert M., Jones P., Gibson P.G., Vestbo J. (2016). Treatable Traits: Toward Precision Medicine of Chronic Airway Diseases. Eur. Respir. J..

[90] Morimoto C., Matsumoto H., Nomura N., Sunadome H., Nagasaki T., Sato S., Sato A., Oguma T., Ito I., Kogo M. (2024). Sputum Microbiota and Inflammatory Subtypes in Asthma, COPD, and Its Overlap. J. Allergy Clin. Immunol. Glob..

[91] Bhatt S.P., Rabe K.F., Hanania N.A., Vogelmeier C.F., Bafadhel M., Christenson S.A., Papi A., Singh D., Laws E., Patel N. (2024). Dupilumab for COPD with Blood Eosinophil Evidence of Type 2 Inflammation. N. Engl. J. Med..

[92] Martinez F.J., Curtis J.L., Albert R. (2008). Role of Macrolide Therapy in Chronic Obstructive Pulmonary Disease. Int. J. Chronic Obs. Pulm. Dis..

[93] Tovchigrechko A., Chiang C.-C., Khatry D., Emson C., Singh D., Wedzichia J., Donaldson G., Brightling C., Finch D., Vaarala O. (2019). COPD Phenotypes Revealed by the Integrated Sputum Microbiome and Proteome Analysis in COPDMAP Cohort. Eur. Respir. J..

[94] Ditz B. (2022). Another Step in COPD-Endotyping: Transcriptomic Profiling of the Host and Respiratory Microbiome.

[95] Fantin A., Manera M., Patruno V., Sartori G., Castaldo N., Crisafulli E. (2023). Endoscopic Technologies for Peripheral Pulmonary Lesions: From Diagnosis to Therapy. Life.

[96] Roche N. (2016). Adding Biological Markers to COPD Categorisation Schemes: A Way towards More Personalised Care?. Eur. Respir. J..

[97] Vanetti M., Visca D., Ardesi F., Zappa M., Pignatti P., Spanevello A. (2025). Eosinophils in Chronic Obstructive Pulmonary Disease. Ther. Adv. Respir. Dis..

[98] Regard L., Roche N. (2025). Difficult-to-Treat COPD: From Concept to Practice. Presse Med..

[99] Christenson S.A., Steiling K., van den Berge M., Hijazi K., Hiemstra P.S., Postma D.S., Lenburg M.E., Spira A., Woodruff P.G. (2015). Asthma-COPD Overlap. Clinical Relevance of Genomic Signatures of Type 2 Inflammation in Chronic Obstructive Pulmonary Disease. Am. J. Respir. Crit. Care Med..

[100] Cosio B.G., Palou A., López M., Engonga R., Valera J.L., Toledo-Pons N. (2025). Towards the Integrated Care of COPD, Asthma and Bronchiectasis: Description and Objectives of a Treatable Trait-Based Complex Obstructive Airway Disease Unit. Expert. Rev. Respir. Med..

[101] Segal L.N., Huang Y.J. (2021). Crossing Kingdoms: Host-Microbial Endotyping and the Quest to Understand Treatable Traits in Chronic Obstructive Pulmonary Disease. Am. J. Respir. Crit. Care Med..

[102] Ryu M.H., Yun J.H., Morrow J.D., Saferali A., Castaldi P., Chase R., Stav M., Xu Z., Barjaktarevic I., Han M. (2023). Blood Gene Expression and Immune Cell Subtypes Associated with Chronic Obstructive Pulmonary Disease Exacerbations. Am. J. Respir. Crit. Care Med..

[103] Crisafulli E., Alfieri V., Silva M., Aiello M., Tzani P., Milanese G., Bertorelli G., Sverzellati N., Chetta A. (2016). Relationships between Emphysema and Airways Metrics at High-Resolution Computed Tomography (HRCT) and Ventilatory Response to Exercise in Mild to Moderate COPD Patients. Respir. Med..

[104] Wang F., Li S., Gao Y., Li S. (2024). Computed Tomography-Based Artificial Intelligence in Lung Disease—Chronic Obstructive Pulmonary Disease. MedComm—Future Med..

[105] Wu Y., Xia S., Liang Z., Chen R., Qi S. (2024). Artificial Intelligence in COPD CT Images: Identification, Staging, and Quantitation. Respir. Res..

[106] Wang M., Li L., Feng M., Liu Z. (2025). Advances in Artificial Intelligence Applications for the Management of Chronic Obstructive Pulmonary Disease. Front. Med..

[107] Awan H.A., Chaudhary M.F.A., El-Sokkary A.D., Hoffman E.A., Comellas A.P., Guo J., Barjaktarevic I.Z., Barr R.G., Bhatt S.P., Bodduluri S. (2025). Association of Lung Quantitative CT Scan Textures With Systemic Inflammation and Mortality in COPD. Chest.

[108] Pinheira A., Casal-Guisande M., Represas-Represas C., Torres-Durán M., Comesaña-Campos A., Fernández-Villar A. (2025). Artificial Intelligence Applications in Chronic Obstructive Pulmonary Disease: A Global Scoping Review of Diagnostic, Symptom-Based, and Outcome Prediction Approaches. Biomedicines.

[109] Budai B.K., Stackelberg O., Kontogianni K., Herth F., Brock J. (2025). Body Composition in COPD Patients with a New AI-Based Radiological Method Compared to Bioelectrical Impedance Analysis. Eur. Respir. J..

[110] Sanders K.J.C., Kneppers A.E.M., van de Bool C., Langen R.C.J., Schols A.M.W.J. (2016). Cachexia in Chronic Obstructive Pulmonary Disease: New Insights and Therapeutic Perspective. J. Cachexia Sarcopenia Muscle.

[111] De Brandt J., Beijers R.J.H.C.G., Chiles J., Maddocks M., McDonald M.-L.N., Schols A.M.W.J., Nyberg A. (2022). Update on the Etiology, Assessment, and Management of COPD Cachexia: Considerations for the Clinician. Int. J. Chronic Obs. Pulm. Dis..

[112] Yevle D.V., Mann P.S., Kumar D. (2026). AI Based Advances in Diagnosis of Chronic Obstructive Pulmonary Disease: A Systematic Review. Comput. Sci. Rev..

[113] Lynch D.A., Austin J.H.M., Hogg J.C., Grenier P.A., Kauczor H.-U., Bankier A.A., Barr R.G., Colby T.V., Galvin J.R., Gevenois P.A. (2015). CT-Definable Subtypes of Chronic Obstructive Pulmonary Disease: A Statement of the Fleischner Society. Radiology.

[114] Occhipinti M., Paoletti M., Bigazzi F., Camiciottoli G., Inchingolo R., Larici A.R., Pistolesi M. (2018). Emphysematous and Nonemphysematous Gas Trapping in Chronic Obstructive Pulmonary Disease: Quantitative CT Findings and Pulmonary Function. Radiology.

[115] Awan H.A., Chaudhary M.F.A., Reinhardt J.M. (2025). Seeing Is Believing-On the Utility of CT in Phenotyping COPD. Br. J. Radiol..

[116] Tisi S., Dickson J.L., Horst C., Quaife S.L., Hall H., Verghese P., Gyertson K., Bowyer V., Levermore C., Mullin A.-M. (2022). Detection of COPD in the SUMMIT Study Lung Cancer Screening Cohort Using Symptoms and Spirometry. Eur. Respir. J..

[117] Robertson N.M., Centner C.S., Siddharthan T. (2024). Integrating Artificial Intelligence in the Diagnosis of COPD Globally: A Way Forward. Chronic Obstr. Pulm. Dis..

[118] Yang X. (2024). Application and Prospects of Artificial Intelligence Technology in Early Screening of Chronic Obstructive Pulmonary Disease at Primary Healthcare Institutions in China. Int. J. Chronic Obs. Pulm. Dis..

[119] Sait A.R.W., Shaikh M.A. (2025). Artificial Intelligence-Powered Chronic Obstructive Pulmonary Disease Detection Techniques-A Review. Diagnostics.

[120] Christenson S.A. (2023). COPD Phenotyping. Respir. Care.

[121] Su L.-F., Zhao H.-M., Xiao Y. (2022). Advances and Application of Genomics in Chronic Obstructive Pulmonary Disease. Zhongguo Yi Xue Ke Xue Yuan Xue Bao.

[122] Ntenti C., Misirlis T.N., Goulas A. (2025). Pharmacogenetic Factors Shaping Treatment Outcomes in Chronic Obstructive Pulmonary Disease. Genes.

[123] Lackey L., McArthur E., Laederach A. (2015). Increased Transcript Complexity in Genes Associated with Chronic Obstructive Pulmonary Disease. PLoS ONE.

[124] Zhuang Y., Hobbs B.D., Hersh C.P., Kechris K. (2021). Identifying miRNA-mRNA Networks Associated With COPD Phenotypes. Front. Genet..

[125] Yu J., Xiao T., Pan Y., He Y., Tan J. (2025). Single Cell Transcriptomics Genomics Based on Machine Learning Algorithm: Constructing and Validating Neutrophil Extracellular Trap Gene Model in COPD. Int. J. Gen. Med..

[126] Li C.-X., Wheelock C.E., Sköld C.M., Wheelock Å.M. (2018). Integration of Multi-Omics Datasets Enables Molecular Classification of COPD. Eur. Respir. J..

[127] Gregory A., Xu Z., Pratte K., Lee S., Liu C., Chase R., Yun J., Saferali A., Hersh C.P., Bowler R. (2022). Clustering-Based COPD Subtypes Have Distinct Longitudinal Outcomes and Multi-Omics Biomarkers. BMJ Open Respir. Res..

[128] Naz S., Kolmert J., Yang M., Reinke S.N., Kamleh M.A., Snowden S., Heyder T., Levänen B., Erle D.J., Sköld C.M. (2017). Metabolomics Analysis Identifies Sex-Associated Metabotypes of Oxidative Stress and the Autotaxin-lysoPA Axis in COPD. Eur. Respir. J..

[129] Prokić I., Lahousse L., de Vries M., Liu J., Kalaoja M., Vonk J.M., van der Plaat D.A., van Diemen C.C., van der Spek A., Zhernakova A. (2020). A Cross-Omics Integrative Study of Metabolic Signatures of Chronic Obstructive Pulmonary Disease. BMC Pulm. Med..

[130] Li B., Liu J., Cao Y., Wang Y., Wu S., Hu H., Xiao X., Hu J., Wang Q., Wu J. (2025). Multi-Omics Characterization of Early Chronic Obstructive Pulmonary Disease. Respir. Res..

[131] Gao J., Liu H., Wang X., Wang L., Gu J., Wang Y., Yang Z., Liu Y., Yang J., Cai Z. (2022). Associative Analysis of Multi-Omics Data Indicates That Acetylation Modification Is Widely Involved in Cigarette Smoke-Induced Chronic Obstructive Pulmonary Disease. Front. Med..

[132] Yan Z., Chen B., Yang Y., Yi X., Wei M., Ecklu-Mensah G., Buschmann M.M., Liu H., Gao J., Liang W. (2022). Multi-Omics Analyses of Airway Host-Microbe Interactions in Chronic Obstructive Pulmonary Disease Identify Potential Therapeutic Interventions. Nat. Microbiol..

[133] Gao J., Yi X., Wang Z. (2023). The Application of Multi-Omics in the Respiratory Microbiome: Progresses, Challenges and Promises. Comput. Struct. Biotechnol. J..

[134] Agusti A., Faner R. (2025). Personalizing COPD Care: Phenotypes, Endotypes, GETomics, the Trajectome, Syndemics and Treatable Traits. Presse Med..

[135] Moll M., Silverman E.K. (2024). Precision Approaches to Chronic Obstructive Pulmonary Disease Management. Annu. Rev. Med..

[136] Sidhaye V.K., Nishida K., Martinez F.J. (2018). Precision Medicine in COPD: Where Are We and Where Do We Need to Go?. Eur. Respir. Rev..

[137] Konigsberg I.R., Vargas L.B., Pratte K.A., Guzman D.E., Pottinger T.D., Buschur K.L., Blackwell T.W., Liu Y., Taylor K.D., Johnson W.C. (2025). Omic Risk Scores Are Associated with COPD-Related Traits Across Three Cohorts. medRxiv.

[138] Zhuang Y., Xing F., Ghosh D., Hobbs B.D., Hersh C.P., Banaei-Kashani F., Bowler R.P., Kechris K. (2023). Deep Learning on Graphs for Multi-Omics Classification of COPD. PLoS ONE.

[139] Hobbs B.D., Morrow J.D., Wang X.-W., Liu Y.-Y., DeMeo D.L., Hersh C.P., Celli B.R., Bueno R., Criner G.J., Silverman E.K. (2023). Identifying Chronic Obstructive Pulmonary Disease from Integrative Omics and Clustering in Lung Tissue. BMC Pulm. Med..

[140] Wang F., Barrero C.A. (2024). Multi-Omics Analysis Identified Drug Repurposing Targets for Chronic Obstructive Pulmonary Disease. Int. J. Mol. Sci..

[141] Wu B.G., Segal L.N. (2018). The Road to Precision Medicine in Chronic Obstructive Pulmonary Disease: Squeezing More Out of Chest Computed Tomography Scans. Ann. Am. Thorac. Soc..

[142] Washko G.R., Parraga G. (2018). COPD Biomarkers and Phenotypes: Opportunities for Better Outcomes with Precision Imaging. Eur. Respir. J..

[143] Sciurba F.C., Criner G.J., Christenson S.A., Martinez F.J., Papi A., Roche N., Bourbeau J., Korn S., Bafadhel M., Han M.K. (2025). Mepolizumab to Prevent Exacerbations of COPD with an Eosinophilic Phenotype. N. Engl. J. Med..

[144] Huang Y., Niu Y., Wang X., Li X., He Y., Liu X. (2024). Identification of Novel Biomarkers Related to Neutrophilic Inflammation in COPD. Front. Immunol..

[145] Bajpai J., Kant S., Matera M.G., Cazzola M. (2025). Targeting Neutrophilic Inflammation in Obstructive Airway Disease—A Narrative Review of Brensocatib Therapy. Respir. Med..

[146] van Zelst C.M., Goossens L.M.A., Witte J.A., Braunstahl G.-J., Hendriks R.W., Rutten-van Molken M.P.M.H., Veen J.C.C.M.I. (2022). Stratification of COPD Patients towards Personalized Medicine: Reproduction and Formation of Clusters. Respir. Res..

[147] Wang J.M., Ram S., Labaki W.W., Han M.K., Galbán C.J. (2022). CT-Based Commercial Software Applications: Improving Patient Care Through Accurate COPD Subtyping. Int. J. Chronic Obs. Pulm. Dis..

[148] Nauck S., Pohl M., Jobst B.J., Melzig C., Meredig H., Weinheimer O., Triphan S., von Stackelberg O., Konietzke P., Kauczor H.-U. (2024). Phenotyping of COPD with MRI in Comparison to Same-Day CT in a Multi-Centre Trial. Eur. Radiol..

[149] Stolz D., Mkorombindo T., Schumann D.M., Agusti A., Ash S.Y., Bafadhel M., Bai C., Chalmers J.D., Criner G.J., Dharmage S.C. (2022). Towards the Elimination of Chronic Obstructive Pulmonary Disease: A Lancet Commission. Lancet.

